# Development and Validation of Fear of Relapse Scale for Relapsing-Remitting Multiple Sclerosis: Understanding Stressors in Patients

**DOI:** 10.3389/fpsyt.2020.00226

**Published:** 2020-03-20

**Authors:** Ali Khatibi, Nahid Moradi, Naghmeh Rahbari, Taranom Salehi, Mohsen Dehghani

**Affiliations:** ^1^Centre of Precision Rehabilitation for Spinal Pain (CPR Spine), School of Sport, Exercise and Rehabilitation Sciences, University of Birmingham, Birmingham, United Kingdom; ^2^Centre for Human Brain Health, University of Birmingham, Birmingham, United Kingdom; ^3^Department of Neurology, Royal Melbourne Hospital, Melbourne, VIC, Australia; ^4^Department of Cognitive Psychology, Institute for Cognitive Science Studies, Tehran, Iran; ^5^Department of Psychology, Allameh Tabatabaei University, Tehran, Iran; ^6^Department of Psychology, Shahid Beheshti University, Tehran, Iran; ^7^Neuroepidemiology Unit, School of Population and Global Health, University of Melbourne, Melbourne, VIC, Australia

**Keywords:** fear, relapse, scale development, psychometric properties, Relapsing-Remitting multiple sclerosis

## Abstract

Chronic diseases are associated with patients’ long-term stress and development of fear to things related to the source of stress. Better management of a patients’ condition requires investigation of the underlying mechanisms that contribute to the process of development of chronic stress. Multiple Sclerosis (MS) is a debilitating chronic disease in most cases diagnosed after a relapse and characterized by the periodic occurrence of relapses in most patients. Due to the unpredictable course of the disease and relapses, patients with Relapsing-Remitting MS (RRMS) may deal with the stress of anticipation of relapse and its unpredictable consequences. The role of relapses and related stress on patients’ quality of life has not been previously investigated. This study is the first effort to develop a self-report measure of Fear of Relapse (FoR) in patients with RRMS. Thirty-one items were extracted from in-depth clinical interviews with 33 RRMS patients to develop the preliminary version of the scale. Subsequently, 168 RRMS patients completed the questionnaire, the Intolerance of Uncertainty Scale (IUS) and Depression, Anxiety, and Stress Scale (DASS). Fifty-one patients completed the scale one more time a month later. Factor analysis revealed three components, and five items failed to load on any of them. To test the FoR’s independence from similar measures, responses to 26 items were pooled once with DASS items and once with IUS items, and each time were subjected to confirmatory factor analysis (two-component solution). Despite significant correlations between FoR, DASS, and IUS Independent loadings of items belonging to FoR and DASS, and FoR and IUS revealed independence and unique contribution of FoR to the evaluation of patients. Cronbach’s alpha for the 26-item version was 0.92. Test-retest reliability for total score was equal to 0.74. These findings provide preliminary evidence of the validity and reliability of the measure. This scale can help researchers and clinicians to have a more comprehensive understanding of patients’ experience with the uncertain nature of MS, which is necessary for future efforts to address this stressor by targeting the underlying mechanism.

## Introduction

Multiple sclerosis is a chronic and potentially debilitating disease of the nervous system that affects people of all ages. Early symptoms vary and may depend on one’s age, life history, and other factors. Diagnosis normally follows the first major occurrence of sub-acute neurological symptoms, which include but are not limited to vision problems, sensory-motor deficits, fatigue, balance problems, and dizziness. The progress of the disease depends on many factors based on which the disease phenotype can be divided into three subgroups, Relapsing-Remitting MS (RRMS), Secondary-Progressive MS (SPMS), and Primary-Progressive MS (PPMS). The course of the disease is unpredictable for individuals, but the majority of patients manifest the relapsing phenotype (85% RRMS) (1). The relapses emerge as neurological events, which may be similar to the first attack after which the disease was diagnosed, or they might be completely different as new areas of the nervous system become involved. The underlying mechanism in the relapsing phenotype is the active inflammation and subsequent demyelination in the Central Nervous System (CNS). These relapses have short and long-term consequences and might lead to the accumulation of disability.

Many previous studies have shown that anxiety is an inseparable part of life in patients with chronic diseases (2). Fear is at the core of anxious thought of patients with chronic diseases, and the source of fear depends on the underlying condition. For example, in chronic pain, fear of pain is due to the patient’s expectations about the influence of pain on his/her personal and social life and how long-term pain causes disability in both (3). Fear of progression has been found to be the pillar of the anxiety problems of patients suffering from chronic diseases like cancer and diabetes mellitus (4–6). A previous effort to develop a tool for measuring fear of progression in chronically ill patients resulted in FoP-Q, which has been shown to have proper psychometric properties (5).

A source of anxiety in the relapsing stages of multiple sclerosis is the relapse itself. Patients diagnosed with these phenotypes do not know when they might have their next relapse and do not have any estimation about the severity of the relapse and its consequences. Generally, it is well-accepted that a new relapse is a sign of disease activity and has its implications such as treatment failure or accumulation of disability. It has also been suggested that fear of progression (as an emotional response) is related to the depression observed in patients with MS while it is not correlated with gender, age, disease duration, and the stage of the disease (7).

Individual prognosis differs among patients with MS. This diversity is partly related to the phenotype and the disease duration, but these alone fail to explain the observed variation. It is important to realize that each MS phenotype is characterized by specific complications and stressors, albeit the fact that demyelination in the CNS might be present throughout the course (8). Any tool that wants to give a clear image in such a clinically diverse disease course must consider these differing stressors. Existing self-report measures describing the psychological impacts of MS on patients are limited in this regard. It has been suggested that both psychological and pharmaceutical interventions are necessary to improve the quality of life in patients with MS (9). The measures that can assess the fear related to the disease activity and the relapses are paramount as they enable evaluating the efficiency of the proposed interventions. We firstly aimed to develop a new tool to measure the fear of relapse in patients with RRMS. Secondly, we aimed to investigate the psychometric properties of this measure in an independent sample of patients.

## Method

Diagnosis of MS in this study is done by a neurologist and based on the 2010 revision of McDonald Criteria (10).

### Interviews and Item Extraction

A semi-structured in-depth interview was conducted with 33 (18 females, age between 24–47 years, mean age = 33.18 ± 6.2 years; disease duration between 3 and 8 years, mean diseases duration = 6.71 ± 3.3 years) patients with RRMS. All the interviews were conducted by the same person (N.R.). Patients were recruited from Sina specialized MS research and treatment center, Tehran, Iran. For all patients, Persian was their native language, and they had received their diagnosis in the previous five years. They were all receiving disease-modifying treatments. Expanded Disability Status Scale score for all these patients was below 4. The study was approved by the ethics committee of the department of psychology at Shahid Beheshti University, and all subjects gave written consent prior to their participation.

The interview was based on pre-selected questions in four main domains: (i) demographics and daily life experiences and difficulties, (ii) cognitive problems, (iii) psychological problems, (iv) social challenges. The interviews took 60 min at most. The sessions were audio-recorded and transcripted afterward and ATLAS.TI-6 was used for the organization of the interview material.

A number of keywords that the literature suggested for individuals’ fear of relapse in MS were selected. This list included: physical difficulties, stress, anxiety, relapse, hospitalization, disease progress, fear, worries, and health problems. A total number of 156 statements were found to contain one or more of these keywords. The content of all 156 items was reviewed by three independent experts and those with similar statements, unclear complaints, and non-relevant content were removed and summarized. The final list contained 31 items judged by their clarity, difficulty in understanding, and relevance. Each item described the patient’s thoughts about symptoms that could be associated with relapse or its consequences. A patient would be asked to mention the degree to which these thoughts came to his/her mind on a five-point Likert scale (0 = Never, 1 = Rarely, 2 = Sometimes, 3 = Often, 4 = Always). The pre-final version of the scale was presented to 10 patients, and their understanding of each item was discussed. We also checked the items with neurologists with expertise in MS and also with psychologists with a focus on the treatment of patients with chronic diseases. As a result, the wording of a few items was adjusted to increase the understandability of them.

An English translation of items is provided in the tables in this article. Items were translated from Persian to English by a bilingual expert, and then back-translated from English to Persian by an independent, experienced translator. Items of the original Persian version and the back-translated items were compared, and the translation of a few items was adjusted accordingly.

### Psychometric Evaluation

#### Participants

A total number of 168 patients with RRMS (142 females; age 17–57 years; mean = 32.65 ± 8.2) were recruited from multiple centers (Golbooteh Omid charity center, Firoozgar hospital, Sina MS research center) in Tehran, Iran, through convenience sampling method. The duration of the disease (since the initial diagnosis by a specialist) was between 0.5 and 5 years (mean = 3.19 ± 1.5) and the patients experienced between 1 and 9 relapses (mean = 3.19 ± 2.2). The last major relapse that resulted in receiving corticosteroid treatment was between 1 and 37 months ago (mean = 12.42 ± 10.6). These patients received either a paper version of the questionnaire or a link to the online version. There was no difference in the outcome of self-report measures and demographics between the groups that completed the paper or the online version. The study was approved by the ethics committee of the Department of Psychology at Shahid Beheshti University and participants had given informed consent prior to participation. For participants younger than 18 years consent was obtained from one of their parents.

### Other Self-Report Measures

The following measures have consistently used in the literature of research on patients with chronic disease to capture the general anxiety and anxiety of an unpredictable future. We included them to test the construct validity of our measure.

#### Depression Anxiety Stress Scale (DASS-21)

DASS-21 (11) is a self-report measure of depression, anxiety, and stress among clinical and nonclinical populations and is reported to have good to excellent psychometric properties in different settings and populations (12, 13). Each item comprises a statement about feelings and experiences that the reader had to select to what degree it applied to them in the past week on a four-point Likert scale (0 = Did not apply to me at all; 3 = Applied to me very much, or most of the time). This measure and the 42 item variant has been translated to Persian and is widely used in different studies and has shown good to excellent psychometric properties [Cronbach’s alpha >0.8; (14, 15)].

#### Intolerance of Uncertainty (IUS-27)

IUS-27 (16) is a self-report measure, with 17 items describing people’s reactions to uncertainties in life. Subjects have to rate each item on a five-point Likert scale (1 = Not at all characteristic of me; 5 = Entirely characteristic of me). This measure is suggested to capture the fear of the unknown and anxiety related to that and is proved to have good psychometric properties (17). This measure has a two-factor structure that is also considered in scoring: a factor that express unfairness of beliefs related to the uncertainty about future and another factor with items about the behavioral and self-referent implications of uncertainty. The Persian version of this scale is widely used in different settings and is showed to have good psychometric properties (internal reliability = 0.88) (18).

### Procedure

After receiving information about the nature of the research and the aim of the current study, participants received either the paper copy of the study or a link to complete the questionnaire online. Fifty-one patients agreed to complete the questionnaire one month later to test the reliability of the measure. The Intolerance of Uncertainty measure, which has been suggested to capture the fear of unknown happening, has been selected to test the construct validity of the fear of relapse measure. For all analyses, the statistical significance level was set to p < 0.05.

## Results

### Statistical Analysis

**Figure 1:**
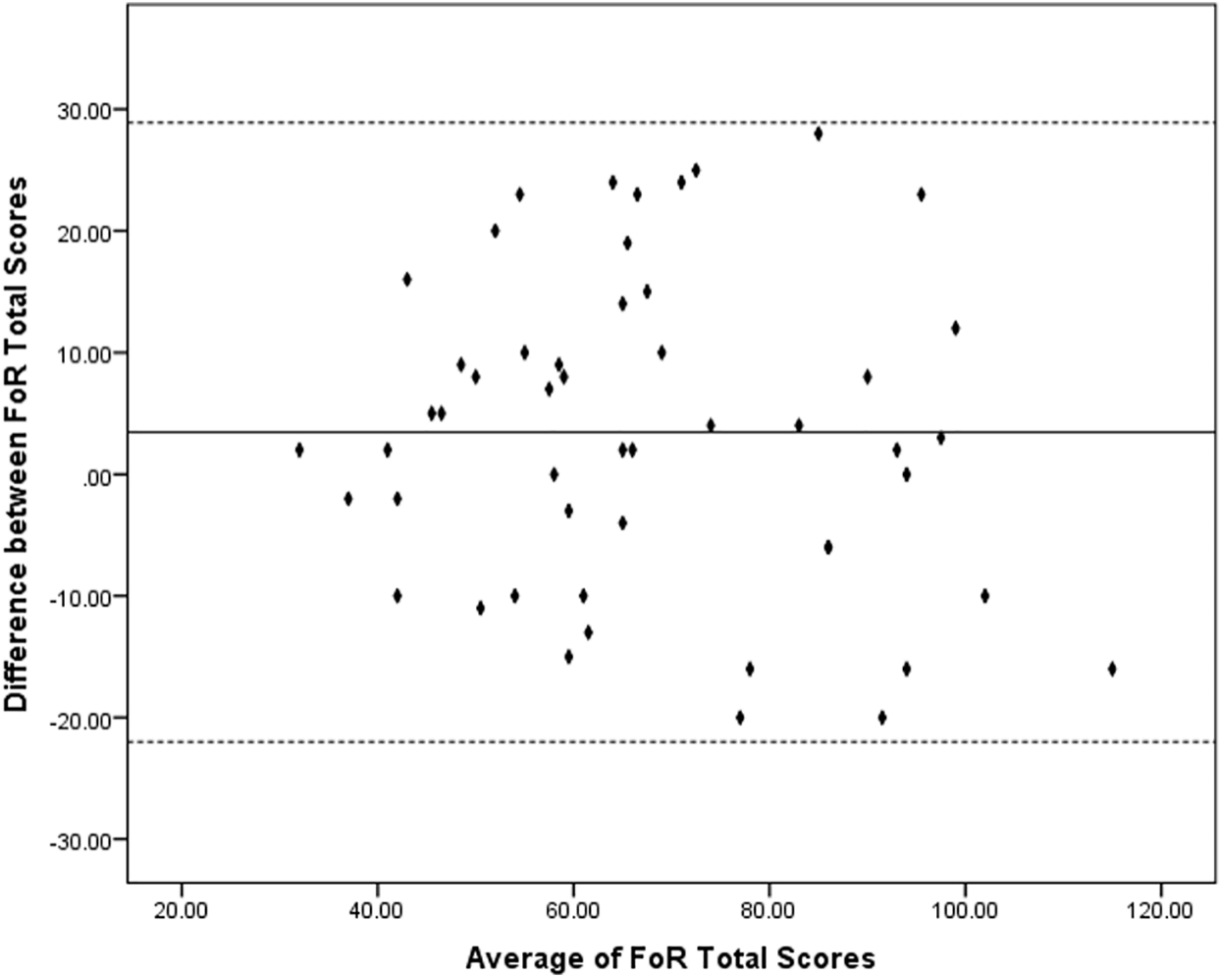
Bland-Altman plot: differences between two times completion of the Fear of Relapse (FoR) Scale by the average of the two scores. Dotted lines indicate the interval that includes 95% of differences between the first and the second assessment.

The frequency and percentage and number of missing responses for each item has been reported. Each item’s correlation with the total score and the Cronbach’s alpha coefficient has been calculated for all items to study the internal consistency of the measure. Correlation analysis between FoR’s score and DASS and IUS was calculated to test the external validity of the scale. The test-retest reliability has been examined using a Spearman Correlation. A Bland-Altman plot (**Figure 1**) was used to display the difference between two times FoR completion and against the average of them. Confirmatory Factor analysis has been hired to examine the internal validity of the scale. To test the external validity and for the convergent validity, the correlation between FoR, DASS, and IUS has been reported. For the discriminant validity, pooled item factor analysis has been performed and the average variance extracted was compared to the square of correlation.

### Depression, Anxiety, and Stress

Based on the DASS scores, patients’ depression was between 0 and 20 with the mean = 8.30 ± 6.0 which is in normal range. None of patients had severe depression and was not excluded from analysis. Anxiety was between 0 and 14 with the mean = 6.90 ± 4.4 which is in normal range. None of patients had severe anxiety and was not excluded from analysis. Stress was between 1 and 21 with the mean = 10.86 ± 5.3 which is in normal range. None of patients had severe stress and was not excluded from analysis.

### Properties of the Fear of Relapse Scale

Responses to 31 items of the Fear of Relapse Scale are summarized in **Table 1**. Total FoR score were between 0 and 113 (mean = 41.6, SD = 21.1). The total score did not follow a normal distribution and showed skewness to the right. In addition, there was no correlation between the FoR total score and the duration of the disease or the number of relapses or time since their last relapse.

**Table 1:** Number of responses for all items of the fear of relapse scale (percentage).

Items	Never	Rarely	Sometimes	Often	Always	Missing
1. I feel another relapse is about to happen whenever I get red eyes or feel pain behind my eyes.	73 (43.5)	42 (25)	28 (16.7)	15 (8.9)	9 (5.4)	1
2. Another relapse means another hospitalization	61 (36.3)	28 (16.7)	41 (24.4)	17 (10.1)	19 (11.3)	2
3. My appearance gives away the fact that I am experiencing a relapse.	68 (40.5)	38 (22.6)	33 (19.6)	16 (9.5)	10 (6)	3
4. Each relapse means the disease is spreading in the nervous system.	34 (20.2)	40 (23.8)	52 (31)	25 (14.9)	13 (7.7)	4
5. I do a lot of exercises because I am afraid of experiencing a relapse.	67 (39.9)	41 (24.4)	28 (16.7)	19 (11.3)	12 (7.1)	1
6. I don’t drive in fear of a relapse.	111 (66.1)	11 (6.5)	14 (8.3)	9 (5.4)	21 (12.5)	2
7. Whenever a relapse happens, it can only be managed with more corticosteroids.	57 (33.9)	23 (13.7)	35 (20.8)	26 (15.5)	25 (14.9)	2
8. The disease will come back in the form of a relapse if I stop taking medication for one month.	50 (29.8)	34 (20.2)	32 (19)	17 (10.1)	32 (19)	3
9. Each relapse takes me one step closer to becoming bedridden.	52 (31)	37 (22)	38 (22.6)	24 (14.3)	17 (10.1)	0
10. Each relapse will make me more dependent on other people.	48 (28.6)	33 (19.6)	29 (17.3)	29 (17.3)	28 (16.7)	1
11. Thinking about relapses makes my heart jitter.	42 (25)	35 (20.8)	40 (23.8)	27 (16.1)	23 (13.7)	1
12. After each relapse, I put all my task and duties aside.	60 (35.7)	49 (29.2)	33 (19.6)	16 (9.5)	8 (4.8)	2
13. A severe relapse with strong symptoms can result in death.	115 (68.5)	24 (14.3)	20 (11.9)	6 (3.6)	3 (1.8)	0
14. Any experience of numbness and tingling in my limbs means I am having another relapse.	47 (28)	51 (30.4)	40 (23.8)	17 (10.1)	13 (7.7)	0
15. Heat can trigger a relapse.	26 (15.5)	39(23.2)	39 (23.2)	38 (22.6)	26 (15.5)	0
16. Relapses cause memory decline.	47 (28)	31 (18.5)	44 (26.2)	30 (17.9)	14 (8.3)	2
17. Relapses cause loss of control over movement and posture stability.	32 (19)	36 (21.4)	39 (23.2)	36 (21.4)	24 (14.3)	1
18. When I think about relapse, I am unable to think about anything else.	43 (25.6)	47 (28)	29 (17.3)	21 (12.5)	25 (14.9)	3
19. Grave news can trigger a relapse.	20 (11.9)	33 (19.6)	54 (32.1)	39 (23.2)	20 (11.9)	2
20. Due to fear of a sudden relapse, I try not to take a shower when I am home alone.	143 (85.1)	13 (7.7)	6 (3.6)	3 (1.8)	3 (1.8)	0
21. Relapses worsen the level of fatigue I feel.	25 (14.9)	27 (16.1)	46 (27.4)	38 (22.6)	31 (18.5)	1
22. Relapses can cause urine and stool incontinence.	78 (46.4)	27 (16.1)	33 (19.6)	18 (10.7)	8 (4.8)	4
23. I try not to go out much due to the fear of experiencing a sudden relapse.	118 (70.2)	13 (7.7)	18 (10.7)	11 (6.5)	6 (3.6)	2
24. Thinking about the disease decreases my libido significantly.	83 (49.4)	28 (16.7)	28 (16.7)	13 (7.7)	15 (8.9)	1
25. I don’t accept new tasks due to fear of relapses.	97 (57.7)	20 (11.9)	23 (13.7)	18 (10.7)	8 (4.8)	2
26. A bad headache can be a sign of a sudden relapse.	64 (38.1)	51 (30.4)	30 (17.9)	11 (6.5)	9 (5.4)	3
27. Whenever I drop anything, I think I am about to have a relapse.	69 (41.1)	41 (24.4)	27 (16.1)	19 (11.3)	12 (7.1)	0
28. The thought of experiencing a relapse makes me cry.	67 (39.9)	27 (16.1)	22 (13.1)	19 (11.3)	32 (19)	1
29. Not knowing when the next relapse is going to happen is very annoying to me.	83 (49.4)	24 (14.3)	18 (10.7)	17 (10.1)	24 (14.3)	2
30. I think increased sensitivity to exercises or tastes can be a sign of relapse.	129 (76.8)	21 (12.5)	10 (6)	6 (3.6)	2 (1.2)	0
31. Blurred vision or double vision can be a sign of relapse.	16 (9.5)	26 (15.5)	45 (26.8)	41 (24.4)	40 (23.8)	0

### Internal Consistency

Each item was correlated with the total score with a correlation value greater than 0.366 (ps <0.001) (**Table 2**). Cronbach’s Alpha was equal to 0.924.

**Table 2:** Internal consistency of the Fear of Relapse Scale.

Item	Item-total correlation	Score (mean ± SD)
1. I feel another relapse is about to happen whenever I get red eyes or feel pain behind my eyes.	.366**	1.07 (1.2)
2. Another relapse means another hospitalization	.541**	1.43 (1.4)
3. My appearance gives away the fact that I am experiencing a relapse.	.521**	1.16 (1.2)
4. Each relapse means the disease is spreading in the nervous system.	.594**	1.65 (1.2)
5. I do a lot of exercises because I am afraid of experiencing a relapse.	.370**	1.21 (1.3)
6. I don’t drive in fear of a relapse.	.453**	0.9 (1.5)
7. Whenever a relapse happens, it can only be managed with more corticosteroids.	.564**	1.63 (1.5)
8. The disease will come back in the form of a relapse if I stop taking medication for one month.	.601**	1.68 (1.5)
9. Each relapse takes me one step closer to becoming bedridden.	.622**	1.51 (1.3)
10. Each relapse will make me more dependent on other people.	.575**	1.74 (1.5)
11. Thinking about relapses makes my heart jitter.	.686**	1.72 (1.4)
12. After each relapse, I put all my task and duties aside.	.583**	1.17 (1.2)
13. A severe relapse with strong symptoms can result in death.	.428**	0.56 (1)
14. Any experience of numbness and tingling in my limbs means I am having another relapse.	.513**	1.39 (1.2)
15. Heat can trigger a relapse.	.371**	1.99 (1.3)
16. Relapses cause memory decline.	.548**	1.6 (1.3)
17. Relapses cause loss of control over movement and posture stability.	.664**	1.9 (1.3)
18. When I think about relapse, I am unable to think about anything else.	.742**	1.62 (1.4)
19. Grave news can trigger a relapse.	.635**	2.04 (1.2)
20. Due to fear of a sudden relapse, I try not to take a shower when I am home alone.	.305**	0.27 (0.8)
21. Relapses worsen the level of fatigue I feel.	.623**	2.14 (1.3)
22. Relapses can cause urine and stool incontinence.	.453**	1.09 (1.2)
23. I try not to go out much due to the fear of experiencing a sudden relapse.	.499**	0.64 (1.1)
24. Thinking about the disease decreases my libido significantly.	.497**	1.1 (1.3)
25. I don’t accept new tasks due to fear of relapses.	.559**	0.92 (1.3)
26. A bad headache can be a sign of a sudden relapse.	.475**	1.09 (1.2)
27. Whenever I drop anything, I think I am about to have a relapse.	.609**	1.19 (1.3)
28. The thought of experiencing a relapse makes me cry.	.622**	1.53 (1.6)
29. Not knowing when the next relapse is going to happen is very annoying to me.	.650**	1.25 (1.5)
30. I think increased sensitivity to exercises or tastes can be a sign of relapse.	.298**	0.4 (0.8)
31. Blurred vision or double vision can be a sign of relapse.	.460**	2.38 (1.3)

### Test-Retest Reliability

Spearman’s rank coefficient was selected to measure test-retest reliability for items independently. All items showed a high correlation (Spearman’s rank coefficients ranged from 0.349 to 0.815 [ps < 0.05]). A Pearson correlation conducted to measure the test-retest reliability of the total score showed to be significant 0.74 (p < 0.001). **Table S1** presents results of the test-retest correlation analysis. **Figure 1** presents the Bland-Altman plot. The difference between two times completion of the FoR is ranged between 28 and −20 (average 3.45 ± 12.98). All the differences fall within 95% confidence of interval from the mean. Regression analysis with the difference score as the dependent variable and the mean of two times completions as the predictor suggest that there is no proportional bias against the mean (t = −0.927; p = 0.36).

### Factor Analysis

A Principal axis factor with Varimax rotation of item from the Fear of Relapse Scale was conducted on the data gathered from patients with RRMS. An examination of the Kaiser-Meyer Olkin measure of sampling adequacy suggested that the sample was factorable (KMO = 0.851; p < 0.001).

The results of an orthogonal rotation of the solution are shown in **Table 3**. When rotated factors items with loadings less than 0.45 were excluded, the analysis yielded a three-factor solution (five items have been suggested to be removed: 1, 24, 26, 30, 31). Only one item (#27) was loaded on two components. **Table 3** presents the result of the factor analysis. Cronbach’s Alpha after removal of the five items mentioned above went down from 0.924 to 0.917.

**Table 3:** Item factor loading for the final solution on 26 final items of the Fear of Relapse Scale.

Item	Component		
	1	2	3
2. Another relapse means another hospitalization	0.75		
9. Each relapse takes me one step closer to becoming bedridden.	0.719		
10. Each relapse will make me more dependent on other people.	0.712		
11. Thinking about relapses makes my heart jitter.	0.694		
7. Whenever a relapse happens, it can only be managed with more corticosteroids.	0.663		
18. When I think about relapse, I am unable to think about anything else.	0.651		
4. Each relapse means the disease is spreading in the nervous system.	0.615		
12. After each relapse, I put all my task and duties aside.	0.564		
28. The thought of experiencing a relapse makes me cry.	0.547		
8. The disease will come back in the form of a relapse if I stop taking medication for one month.	0.543		
29. Not knowing when the next relapse is going to happen is very annoying to me.	0.542		
27. Whenever I drop anything, I think I am about to have a relapse.	0.495	0.495	
3. My appearance gives away the fact that I am experiencing a relapse.	0.488		
16. Relapses cause memory decline.		0.713	
14. Any experience of numbness and tingling in my limbs means I am having another relapse.		0.659	
15. Heat can trigger a relapse.		0.64	
21. Relapses worsen the level of fatigue I feel.		0.629	
17. Relapses cause loss of control over movement and posture stability.		0.562	
19. Grave news can trigger a relapse.		0.544	
22. Relapses can cause urine and stool incontinence.		0.496	
13. A severe relapse with strong symptoms can result in death.		0.486	
23. I try not to go out much due to the fear of experiencing a sudden relapse.			0.714
5. I do a lot of exercises because I am afraid of experiencing a relapse.			0.708
6. I don’t drive in fear of a relapse.			0.61
20. Due to fear of a sudden relapse, I try not to take a shower when I am home alone.			0.597
25. I don’t accept new tasks due to fear of relapses.			0.531

Five items with loading weights below 0.45 were removed from the table and the final version of the scale.

### Validity

To test conduct validity of the Fear of Relapse Scale we used a Pearson correlation analysis to examine the correlation between the Fear of Relapse Scale (total score after removing five items, three components as the result of factor analysis) and IUS score on two factors and DASS subscales (Depression, Anxiety, Stress). **Table 4** presents the result of the correlation analysis. All the correlations were significant (ps < 0.001).

**Table 4:** Correlations between Depression, Anxiety, Stress, Scale, Intolerance of Uncertainty Scale, Fear of Relapse Scale and its Subscales.

	DASS_D	DASS_A	DASS_S	IUS_F1	IUS_F2
**FoR_total**	.600^**^	.587^**^	.588^**^	.513^**^	.527^**^
**FoR_Comp1**	.540^**^	.497^**^	.542^**^	.491^**^	.495^**^
**FoR_Comp2**	.521^**^	.546^**^	.520^**^	.449^**^	.458^**^
**FoR_Comp3**	.400^**^	.422^**^	.338^**^	.316^**^	.358^**^

DASS_D, Depression subscale of DASS; DASS_A, Anxiety subscale of DASS; DASS_S, Stress subscale of DASS; IUS_F1, Factor 1 of the Intolerance of Uncertainty Scale; IUS_F2, Factor 2 of the Intolerance of Uncertainty Scale.

**p < 0.001.

A two-component solution factor analysis by pooling the 26 items of the FoR and DASS items (principal components, varimax rotation, maximum iterations for convergence = 50, excluding values below 0.35) resulted in a clear separation of two scales on two components. For the DASS only items 4, 15, and 19 weighted below 0.35, and for the FoR, only item 20 weighted below 0.35. For the DASS items 3, 5, and 15 loaded on both components while the weight on the first component related to other DASS items (respectively 0.615, 0.592, 0.707) was much higher than the weight for on the second component related to FoR items (respectively 0.358, 0.376, 0.353). For the FoR, only the item numbered 16 loaded on both components, while the weight for the second component related to FoR items (0.418) was higher than the weight on the first component (0.415). The average variance extracted for both FoR and DASS (0.702) is higher than the square of correlation between FoR and DASS (0.589). Hence discriminant validity is established.

A similar analysis using the IUS items instead of the DASS resulted in a clear separation of two scales on two components. For this analysis, only the item numbered 20 of the FoR weighted below 0.35 and was removed. Besides, only two IUS items (1 and 7) were loaded on both components. Both items were loaded on the first component (along with other IUS items; respectively 0.438, 0.608) higher than the second component related (along with FoR items; respectively 0.397, 0.393). The average variance extracted for both FoR and IUS (0.637) is higher than the square of correlation between FoR and IUS (0.498). Hence discriminant validity is established.

## Discussion

The most common phenotype of MS known as relapsing-remitting MS appears in the form of unpredictable relapses that are associated with the progression of the disease and may result in irreversible damages to the sensations and functions of a person. Like other chronic diseases, fear of consequences, worsening, and relapse is a dominant part of MS as well. The nature of fear and the source of fear have not been investigated very well in the literature on MS. Only a few studies compared the patients’ fear of falling between different age groups of MS patients (19). Understanding and the management of fear in chronic diseases have been suggested to be associated with the improvement in self-management in patients (20). Consequently, improved self-management in chronic disease has been suggested to be associated with an improvement in health status, increase in self-efficacy, and a reduction in hospitalization (21). Thus, increased self-management of a chronic disease reduces the economic and emotional burden of the disease and might result in a substantial increase in the quality of life of patients with chronic illnesses (22). Relapses in MS with unknown course and severity are a big source of anxiety in patients and contribute to the development of fear in patients (23). However, as far as we are aware, fear of relapse and its impacts on the quality of life has not been fully addressed, and we need to develop a scale to screen MS patients. Measuring fear of relapse may help the health care system to treat patients in a more personalized approach. As such, the current study aimed to develop a measure that can quantify fear of relapse in patients suffering relapsing-remitting MS and to validate it in a separate sample of participants.

To test the validity of the Fear of Relapse Scale, the patients were asked to complete two other measures assessing their intolerance of uncertainty (fear of unknown) and stress, anxiety, and depression. Both scales and all subscales were significantly and positively correlated with individuals’ total fear of relapse score. The Pearson correlation was at medium range suggesting a relationship between the FoR scale, DASS, and IUS measures. Further investigations, however, showed that when two independent two-components factor analyses were run by pooling items of the Fear of Relapse Scale once with the IUS and once with the DASS, items related to each scale were independently loaded on a separate component, which suggest that in spite of a positive and significant correlation, each of the included measures assess independent constructs. This suggests that inclusion of the fear of relapse in research and clinical work with MS patients can capture characteristics in patients that cannot solely be explained by intolerance of uncertainty, depression, anxiety, and stress in patients.

We tested the reliability based on test-retest and internal consistency, Cronbach’s Alpha. The Cronbach’s alpha for the scale was 0.94, which is above the suggested threshold for the assessment of reliability (24). In line with this analysis, all items in the measure were positively and significantly correlated with the total score of the scale, which indicates the high internal consistency of the Fear of Relapse Scale (25). Furthermore, the test-retest reliability showed a high positive and significant correlation between each item’s score in the test and that item’s score in the retest. Above that, the total score of test-retest reliability showed a high, positive correlation (>0.7). Similarly, the Bland-Altman analysis and the plot indicated that there is a high level of agreement between two times completion of the scale and there is no trend or proportional bias in differences between two times completion of the scale. It is important to remember that none of the patients experienced a relapse or any major problem related to their disease within the period between two assessments. Putting these results together it can be suggested that the Fear of Relapse Scale is a reliable measure, but further studies with long-term follow-up of patients are required to examine the ability of the scale to capture changes due to the alterations in the condition of the patient.

A principal component analysis of all 31 original items of the Fear of Relapse Scale resulted in the removal of 5 items and the remaining 26 items loaded on three factors. The first factor includes items that refer to the fear of disability following a relapse. Disability is a concern for many patients who receive MS diagnosis. The link between the experience of relapse and the progress of the disease causes patients to see each relapse as another step towards the disability, and this can result in the experience of excessive hypervigilance and fear of relapse. The second factor includes items related to the fear of the psychological and physiological consequences of a relapse. The difference between this component and the previous one is in the understanding of the fact that disability is not the sole outcome of the disease and relapses. A relapse may cause a lot of secondary problems, which can interfere with the life of the patient, and items in the second component target these aspects. Usually, relapses are followed by short-term (or in some cases an irreversible) losses in the function of the sensory and motor systems in the central nervous system. Uncertainty about the prognosis of the sensory and motor losses and the future of patients’ functions is another source of stress feeding their fear of relapse. The third factor includes items indicating limitations resulting from fear. After the diagnosis, many patients become hypervigilant to the potential impact of the disease on their abilities and thus, they may start to withdraw from many activities and stop accepting new challenges. This avoidance may contribute to the development of fear and further disability and lowering self-efficacy. Compared to the other two components, this component relates to the meta-cognition of patients about the existence of fear and its influence on their life.

Fear of relapse in MS is a neglected phenomenon in the studies investigating the psychological aspects of the diagnosis and living with MS. Psychological factors play a crucial role in the quality of life in patients with MS especially due to the chronic nature of the disease (26). A better understanding of a chronic disease requires a comprehensive approach to take different components into account, especially when it comes to the quality of life as a multi-dimensional aspect (27). A previous attempt to develop measures related to the quality of life in MS resulted in the PERSEPP scale (“PERception de la Sclérose En Plaques et de ses Poussées”) (28). Five dimensions of that scale target social support, relationship difficulties, fatigue, state of mind and associated sleep disorders, and time perspective. However, fear as a factor related to the understanding of the patient’s acceptance of has not received in depth attention there. Understanding the source of fear in MS patients will assist professionals to grasp a more detailed image of their life to provide more specific and effective interventions (29). It can be suggested that interventions for the improvement of the fear of relapse might be more effective if provided at the earlier stages of the development of the disease (30).

However, this study has limitations too. The sample of patients in the current study had varying disease duration. Although we did not find any association between the duration of the disease and fear of relapse scores, previous studies suggested that the impact of psychological factors in the first year following the diagnosis is stronger on the quality of life of the patients (30). Also, RRMS patients vary greatly in earlier clinical presentations in comparison with more chronic progressive phenotype (31). Future studies with a larger number of patients in their first year following the diagnosis may help us to characterize fear of relapse among them and to compare them with patients who have experienced more relapses and have a longer disease duration. Besides, since the concept of fear of relapse has not been well investigated in the literature, future studies on a diverse group of patients will allow us to examine the content validity of the measure in more detail. Also, long-term follow-up of patients and changes in their FoR scores can give us a more comprehensive image beside the test-retest reliability. Furthermore, the original version of the scale was developed in Persian. The English translation needs to be investigated in English speaking populations. As such, the measure should be the subject for future studies in another language considering linguistic and cultural differences.

Taken together, this study presents a scale for the assessment of the fear of relapse in RRMS patients. Our data provides preliminary evidence of validity and reliability for this scale. The Fear of Relapse Scale presented in this study showed good internal consistency and high test-retest reliability. This measure needs to be tested in future studies to contribute to the understanding of the life of the patients suffering RRMS and may assist clinicians in identifying the specific sources of anxiety in patients and helping them to plan their interventions tailored to the clinical needs of the patient.

## Data Availability Statement

The datasets generated for this study are available on request to the corresponding author.

## Ethics Statement

The studies involving human participants were reviewed and approved by the ethics committee of the Department of Psychology at Shahid Beheshti University, Tehran, Iran. The patients/participants provided their written informed consent to participate in this study.

## Author Contributions

AK was involved in design, data collection, analysis, and writing. NM was involved in data collection and writing. NR was involved in data collection. TS was involved in data collection. MD was involved in design, analysis, and writing.

## Conflict of Interest

The authors declare that the research was conducted in the absence of any commercial or financial relationships that could be construed as a potential conflict of interest.
