# Supplementary material for: Development and Validation of Fear of Relapse Scale for Relapsing-Remitting Multiple Sclerosis: Understanding Stressors in Patients
Source: Front Psychiatry. 2020 Mar 20;11:226. doi: 10.3389/fpsyt.2020.00226 (PMC7100001; doi:10.3389/fpsyt.2020.00226)
Supplement: Supplementary file 1 [file Table_1.docx]

| **Table S1 Test-retest reliability of the Fear of Relapse Scale** | | | |
| --- | --- | --- | --- |
| **Items** | **Mean_test (SD_test)** | **Mean_reTest (SD_reTest)** | **Spearman's rank coefficient** |
| 1. I feel another relapse is about to happen whenever I get red eyes or feel pain behind my eyes. | 1.07(1.2) | 1.84(1.1) | .702** |
| 2. Another relapse means another hospitalization | 1.43(1.4) | 2.17(1.1) | .553** |
| 3. My appearance gives away the fact that I am experiencing a relapse. | 1.16(1.2) | 2.16(1.3) | .435** |
| 4. Each relapse means the disease is spreading in the nervous system. | 1.65(1.2) | 2.61(1.3) | .539** |
| 5. I do a lot of exercises because I am afraid of experiencing a relapse. | 1.21(1.3) | 1.93(1) | .815** |
| 6. I don’t drive in fear of a relapse. | 0.9(1.5) | 1.61(1.1) | .553** |
| 7. Whenever a relapse happens, it can only be managed with more corticosteroids. | 1.63(1.5) | 1.89(0.9) | .661** |
| 8. The disease will come back in the form of a relapse if I stop taking medication for one month. | 1.68(1.5) | 2.39(1.4) | .469** |
| 9. Each relapse takes me one step closer to becoming bedridden. | 1.51(1.3) | 2.04(1.2) | .561** |
| 10. Each relapse will make me more dependent on other people. | 1.74(1.5) | 2.24(1.3) | .567** |
| 11. Thinking about relapses makes my heart jitter. | 1.72(1.4) | 2.28(1.3) | .478** |
| 12. After each relapse, I put all my task and duties aside. | 1.17(1.2) | 2.04(1.1) | .598** |
| 13. A severe relapse with strong symptoms can result in death. | 0.56(1) | 1.47(0.9) | .620** |
| 14. Any experience of numbness and tingling in my limbs means I am having another relapse. | 1.39(1.2) | 2.22(1.1) | .658** |
| 15. Heat can trigger a relapse. | 1.99(1.3) | 2.76(1.3) | .622** |
| 16. Relapses cause memory decline. | 1.6(1.3) | 2.39(1.3) | .683** |
| 17. Relapses cause loss of control over movement and posture stability. | 1.9(1.3) | 2.48(1.3) | .735** |
| 18. When I think about relapse, I am unable to think about anything else. | 1.62(1.4) | 2.17(1.4) | .605** |
| 19. Grave news can trigger a relapse. | 2.04(1.2) | 2.58(1.2) | .630** |
| 20. Due to fear of a sudden relapse, I try not to take a shower when I am home alone. | 0.27(0.8) | 1.07(0.2) | .635** |
| 21. Relapses worsen the level of fatigue I feel. | 2.14(1.3) | 2.61(1.3) | .688** |
| 22. Relapses can cause urine and stool incontinence. | 1.09(1.2) | 1.83(1) | .560** |
| 23. I try not to go out much due to the fear of experiencing a sudden relapse. | 0.64(1.1) | 1.22(0.6) | .482** |
| 24. Thinking about the disease decreases my libido significantly. | 1.1(1.3) | 1.7(1.1) | .377** |
| 25. I don’t accept new tasks due to fear of relapses. | 0.92(1.3) | 1.67(1) | .395** |
| 26. A bad headache can be a sign of a sudden relapse. | 1.09(1.2) | 1.8(0.9) | .664** |
| 27. Whenever I drop anything, I think I am about to have a relapse. | 1.19(1.3) | 1.83(1.2) | .430** |
| 28. The thought of experiencing a relapse makes me cry. | 1.53(1.6) | 1.98(1.3) | .557** |
| 29. Not knowing when the next relapse is going to happen is very annoying to me. | 1.25(1.5) | 1.72(1.2) | .647** |
| 30. I think increased sensitivity to exercises or tastes can be a sign of relapse. | 0.4(0.8) | 1.33(0.8) | .349* |
| 31. Blurred vision or double vision can be a sign of relapse. | 2.38(1.3) | 3(1.3) | .432** |

**Fear of Relapse Scale-EN**

| Statements presented below, depict the thoughts that may pass a patient’s mind. Please read each item carefully and then mention how often these thoughts may come to your mind. | | | | | | |
| --- | --- | --- | --- | --- | --- | --- |
| **Item** | **Statements** | **Never** | **Rarely** | **Sometimes** | **Often** | **Always** |
| 1** | I feel another relapse is about to happen whenever I get red eyes or feel pain behind my eyes. |  |  |  |  |  |
| 2 | Another relapse means another hospitalization |  |  |  |  |  |
| 3 | My appearance gives away the fact that I am experiencing a relapse. |  |  |  |  |  |
| 4 | Each relapse means the disease is spreading in the nervous system. |  |  |  |  |  |
| 5 | I do a lot of exercises because I am afraid of experiencing a relapse. |  |  |  |  |  |
| 6 | I don’t drive in fear of a relapse. |  |  |  |  |  |
| 7 | Whenever a relapse happens, it can only be managed with more corticosteroids. |  |  |  |  |  |
| 8 | The disease will come back in the form of a relapse if I stop taking medication for one month. |  |  |  |  |  |
| 9 | Each relapse takes me one step closer to becoming bedridden. |  |  |  |  |  |
| 10 | Each relapse will make me more dependent on other people. |  |  |  |  |  |
| 11 | Thinking about relapses makes my heart jitter. |  |  |  |  |  |
| 12 | After each relapse, I put all my task and duties aside. |  |  |  |  |  |
| 13 | A severe relapse with strong symptoms can result in death. |  |  |  |  |  |
| 14 | Any experience of numbness and tingling in my limbs means I am having another relapse. |  |  |  |  |  |
| 15 | Heat can trigger a relapse. |  |  |  |  |  |
| 16 | Relapses cause memory decline. |  |  |  |  |  |
| 17 | Relapses cause loss of control over movement and posture stability. |  |  |  |  |  |
| 18 | When I think about relapse, I am unable to think about anything else. |  |  |  |  |  |
| 19 | Grave news can trigger a relapse. |  |  |  |  |  |
| 20 | Due to fear of a sudden relapse, I try not to take a shower when I am home alone. |  |  |  |  |  |
| 21 | Relapses worsen the level of fatigue I feel. |  |  |  |  |  |
| 22 | Relapses can cause urine and stool incontinence. |  |  |  |  |  |
| 23 | I try not to go out much due to the fear of experiencing a sudden relapse. |  |  |  |  |  |
| 24** | Thinking about the disease decreases my libido significantly. |  |  |  |  |  |
| 25 | I don’t accept new tasks due to fear of relapses. |  |  |  |  |  |
| 26** | A bad headache can be a sign of a sudden relapse. |  |  |  |  |  |
| 27 | Whenever I drop anything, I think I am about to have a relapse. |  |  |  |  |  |
| 28 | The thought of experiencing a relapse makes me cry. |  |  |  |  |  |
| 29 | Not knowing when the next relapse is going to happen is very annoying to me. |  |  |  |  |  |
| 30** | I think increased sensitivity to exercises or tastes can be a sign of relapse. |  |  |  |  |  |
| 31** | Blurred vision or double vision can be a sign of relapse. |  |  |  |  |  |
| ** Items removed from the final version of the scale. | | | | | | |
